# Long chain ionic liquid-assisted synthesis of PS/Pd beads and hierarchical porous Pd–SiO_2_†

**DOI:** 10.1039/c8ra03884h

**Published:** 2018-07-13

**Authors:** Tianlong Wang, Ting Fu, Yuting Meng, Jing Shen, Tongwen Wang

**Affiliations:** Department of Applied Chemistry, College of Vocational Education, Yunnan Normal University Kunming 650092 China shenjingbox0225@hotmail.com; College of Chemistry and Chemical Engineering, Yunnan Normal University Kunming 650092 China

## Abstract

Long-chain ionic liquid, 1-hexadecyl-3-methylimidazolium chloride (C_16_mimCl), was firstly used as a linking agent to construct polystyrene (PS)/C_16_mimCl/palladium (Pd) beads. Subsequently, the PS/C_16_mimCl/Pd beads were used as a macroporous templating agent and C_16_mimCl was used as a mesoporous templating agent to prepare Pd-loaded hierarchical porous silica. A systematic study was carried out addressing the influence of the amount of C_16_mimCl and the mass ratios of *m*(Pd)/*m*(PS) on the PS/C_16_mimCl/Pd beads and the Pd-loaded hierarchical porous structures. The samples were characterized by electrophoresis experiments, SEM, TEM, small-angle XRD, and N_2_ adsorption–desorption experiments. It was found that the coverage of citrate-coated Pd nanoparticles (Pd NPs) onto the PS beads can be simply tailored by changing the amount of C_16_mimCl and the mass ratios of *m*(Pd)/*m*(PS). The resultant Pd-loaded hierarchical porous silica possessed a 3D ordered macroporous skeleton with a specific surface area of up to 967 m^2^ g^−1^, ordered mesoporous silica walls (SBA-3 type) and well-dispersed Pd NPs anchored on the inner walls of the spherical macroporous hollow. Importantly, the obtained Pd-loaded hierarchical porous silica exhibited an enhanced catalytic activity for the oxidation of 3,3′,5,5′-tetramethylbenzidine (TMB) by H_2_O_2_.

## Abstract 2

1-Hexadecyl-3-methylimidazolium chloride (C_16_mimCl) can be used as “bridges” to prepare PS/C_16_mimCl/Pd beads, and Pd-loaded hierarchical porous silica was synthesized using dual templates of the PS/C_16_mimCl/Pd beads and C_16_mimCl.

## Graphical abstract

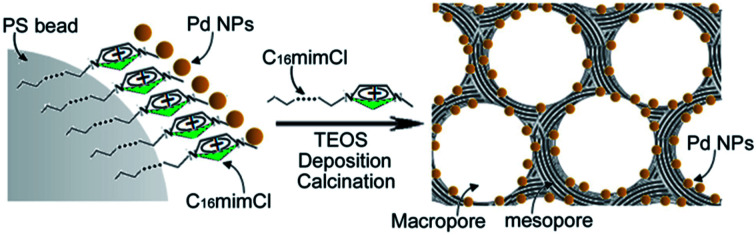

## Introduction

Long-chain ionic liquids (LILs), especially imidazolium-based LILs, have been most extensively studied for both fundamental research and practical applications.^1^ The LILs consist of a charged hydrophilic head group and a hydrophobic ‘tail’ domain, which can display not only the amphiphilic properties of conventional surfactants, but also the specificity of the typical short-chain room temperature ionic liquids such as low melting point, strong polarity and high solubility in aqueous solution *etc.* Increasing efforts have been made to explore the self-organized behavior of the LILs in aqueous solution.^2^ Various characterization methods, such as surface tension, electrical conductivity and fluorescence measurement, have been used to determine the micelle formation and the micelle aggregation numbers of the LILs in aqueous solution.^3–6^ The results indicated that the surface activity of the LILs is slightly superior to that of the typical cationic surfactants. Moreover, the LILs have been demonstrated to exhibit the behavior of both lyotropic and thermotropic liquid crystals,^7,8^ which offer a partially ordered microenvironment for organic reactions.^9^ These advantages of the LIL derivatives have been broadly applied in the introduction of ordered self-organized structures for synthesis of functional nanoparticles and other inorganic nanostructures.^10,11^ Particularly, the supramolecular templating function of the LILs has been used to create various ordered porous materials. For example, Adams *et al.* reported the synthesis of mesoporous silica with 2D hexagonal structure by using the LILs as templating agent.^12^ Zhou *et al.* employed long-chain 1-hexadecyl-3-methylimidazolium chloride (abbreviated as C_16_mimCl), as template to prepare a supermicroporous lamellar silica by so-called “nanocasting” technique.^13,14^ Our group has also contributed much effort to successfully synthesize the mesoporous silica with high-quality cubic gyroid and 2D hexagonal mesoporous structures by using C_16_mimCl as template.^15^ These results indicated that the LILs have significantly stronger tendency toward self-aggregation and supramolecular templating in the preparation of ordered mesoporous structure. However, their potential as modifying agent, especially the formation of LIL-functionalized polymer microspheres or their multifunctional combination for the synthesis of nanostructured materials is less commonly known.

The objective of the present work is twofold: firstly, long-chain ionic liquid, 1-hexadecyl-3-methylimidazolium chloride (C_16_mimCl) was used as a surface functional molecule to modify polystyrene (PS) beads, which can effectively connect small citrated-coated palladium (Pd) nanoparticles (Pd NPs) to construct PS/C_16_mimCl/Pd beads. Secondly, based on a dual templating method of the PS/C_16_mimCl/Pd beads and C_16_mimCl, a more facile access to prepare Pd-loaded silica with hierarchical porous (macropore-mesopore) structures and catalytic activities is presented.

Recently, loading Pd NPs on suitable supports have attracted great attention due to their unique catalytic properties for the carbon–carbon cross-coupling reactions.^16,17^ Coating Pd NPs on polymer beads can not only provide high dispersion and stabilization for small Pd NPs and large surface-to-volume ratio to increase more number of catalytically active sites, but also be easily isolated and recycled from the reaction medium by filtration or centrifugation.^18–20^ The traditional self-assembly approach for the synthesis of polymer/Pd beads is based on preformed Pd NPs sorption on polymer beads. For example, Thompson and co-workers reported an electrostatic deposition of colloidal noble metals (Pt, Pd, and Au) on the surface of PS beads. Before the deposition, the non-cross-linked PS beads were functionalized by using carboxylic acid- and amino-groups to offer an effective connection of small noble-metal NPs.^21^ Here, the C_16_mimCl is designed to modify PS beads, which forms the C_16_mimCl-functionalized PS beads for the deposition of the preformed citrated-coated Pd NPs.

Ordered porous materials can also be considered as an ideal scaffold for loading Pd NPs.^22,23^ Particularly, bimodal mesoporous–macroporous inorganic materials possess increased mass transport through its macropores as well as retaining a specific surface area on the level of fine mesoporous structures.^24^ In previously reported preparation of Pd NPs-loaded ordered porous silica, the commonly used method is simple impregnation with metal salt solutions into porous channels and further reduction to form Pd NPs in the porous silica channels. However, this method often causes the partial plugging and destruction of the porous system because of crystallization of the metal salt during the calcination process to remove the pore-forming templates. Some improved approaches have been reported to enhance the stabilization of the Pd NPs-loaded hierarchical porous structure. For instance, Zhou *et al.* reported the cyclodextrin (CD)-based homogeneous incorporation of Pd NPs into silica with bimodal pore structure.^25^ In the present synthesis of the Pd-loaded hierarchical porous silica, the prepared PS/C_16_mimCl/Pd beads and long-chain C_16_mimCl were employed as dual templates to create 3D macroporous silica skeleton with ordered mesoporous walls. The inverse replicas of the PS/C_16_mimCl/Pd beads resulted in spherical voids, in which Pd NPs were anchored on the inner walls of the spherical voids, and the inverse replicas of the C_16_mimCl leaded to ordered mesoporous silica channel walls. The method that employs the LILs not only as a connector but also as a templating agent in one system should provide important guidelines for the multifunctional applications of the LILs in the synthesis of nanostructured materials.

## Experimental section

### Synthesis of C_16_mimCl

Long-chain ionic liquid, 1-hexadecyl-3-methylimidazolium chloride (C_16_mimCl) was prepared according to a route reported in the literature.^15,26^ All chemicals were purchased from Acrös and used as received. As a typical synthesis, 1-methylimidazole (10.26 g, 0.125 mol) was mixed with an excess of 1-hexadecylchloride (33.41 g, 0.128 mol). The mixture was put into a 250 mL flask, refluxed at 90 °C for 24 h, and then cooled down to room temperature. The product was further purified by recrystallization in tetrahydrofuran (THF). After washing several times with THF, the white crystalline powder of C_16_mimCl was collected by vacuum filtration, and dried in air at room temperature. The structure of the obtained C_16_mimCl was identified by IR spectrum (see Fig. S1†).

### Synthesis of PS/C_16_mimCl beads

Monodisperse PS beads were synthesized according to an emulsion polymerization using sodium dodecylbenzene sulfonate (SDBS) as emulsifier. Typically, 21 mL of styrene was washed in a separatory funnel four times with 20 mL of 0.1 M NaOH, then four times with 20 mL water. 0.005 g of SDBS was dissolved in 180 mL water in a round-bottomed flask. The washed styrene was added, and nitrogen was bubbled to deaerate the mixture for 20 min. 0.2328 g of potassium persulfate initiator was added to the mixture, and the mixture was then heated to 70 °C for 12 h with rapid stirring. The PS beads in emulsion were collected by centrifugation.

The PS bead solid was redispersed in water under ultrasound. 50 mL of C_16_mimCl aqueous solution (2.985 g L^−1^) was added dropwise and slowly to 10 mL of the PS bead dispersion (4.686 g L^−1^) with stirring for 4 h at room temperature to obtain the dispersion (0.781 g L^−1^) of the C_16_mimCl-coated PS beads (PS/C_16_mimCl). In order to explore the effect of the amount of adding C_16_mimCl on the surface charge of the PS/C_16_mimCl beads, the different volumes of the C_16_mimCl solution (*V*(C_16_mimCl) = 1, 25, 75 and 100 mL) were also added dropwise to the PS bead dispersion.

### Synthesis of PS/C_16_mimCl/Pd beads

Pd nanoparticle (Pd NP) dispersion was prepared by using sodium citrate as a reduction agent as well as a stabilizing agent according to a reported method.^27^ Briefly, 7.5 mL of PdCl_2_ solution (0.99 g L^−1^), 15 mL of sodium citrate solution (60 g L^−1^), and 52.5 mL of water were mixed with stirring. The mixture was heated to boil, and kept boiling and stirring for 6 h. The color of the citrate-coated Pd NP (Pd/Cit.NP) dispersion (0.0599 g L^−1^) turned from yellow to brown.

The PS/C_16_mimCl/Pd beads were synthesized using the PS/C_16_mimCl beads as nuclei for subsequent adsorption deposition of Pd/Cit.NPs according to the compositions listed in Table 1. The different volumes of the Pd/Cit.NP dispersion (*V*(Pd/Cit.NP) = 15.62, 31.24, 46.86 and 62.48 mL) (0.0599 g L^−1^) were added dropwise to 20 mL of the PS/C_16_mimCl dispersion (0.781 g L^−1^), respectively. The mixture was sufficiently stirred for 4 h to obtain the PS/C_16_mimCl/Pd beads. The mass ratios of *m*(Pd)/*m*(PS) were calculated based on the mass of Pd in the Pd/Cit.NP dispersion and the mass of PS in the PS/C_16_mimCl dispersion to be 6%, 12%, 18% and 24%, respectively. The four PS/C_16_mimCl/Pd bead samples were coded as PS/C_16_mimCl/Pd(*x*), *x* = 6%, 12%, 18% and 24%, respectively.

**Table 1:** Synthesis recipe of PS/C_16_mimCl/Pd(*x*)

Sample	PS/C_16_mimCl (mL)	Pd/Cit. (mL)
PS/C_16_mimCl/Pd(6%)	20	15.62
PS/C_16_mimCl/Pd(12%)	20	31.24
PS/C_16_mimCl/Pd(18%)	20	46.86
PS/C_16_mimCl/Pd(24%)	20	62.48

### Synthesis of Pd-loaded hierarchical porous silica

The Pd-loaded hierarchical porous silica was prepared in acid medium by a sol–gel procedure using tetraethylorthosilicate (TEOS) as silicon source, the prepared PS/C_16_mimCl/Pd beads and C_16_mimCl as macroporous and mesoporous structural templating agents, respectively. In a typical synthesis procedure, C_16_mimCl and HCl were added to the PS/C_16_mimCl/Pd dispersion under mild magnetic stirring. After homogenization of the mixture, TEOS was added dropwise at room temperature. The molar compositions of the starting mixtures were *n*(TEOS)/*n*(C_16_mimC)/*n*(HCl)/*n*(H_2_O) = 0.23/0.2/56/504. The resulting mixtures were stirred at room temperature for 2 h and then aged for 12 h. After the sol–gel treatment, the mixtures were filtered, washed with deionized water, dried under atmosphere at room temperature, and finally calcined by a temperature programmed route at 300 °C for 2 h, and then, 550 °C for 2 h with a temperature ramp of 2 °C min^−1^ under static air condition to remove the templates. The resultant sample based on the PS/C_16_mimCl/Pd(12%) beads and C_16_mimCl dual templates is coded as Pd–SiO_2_(12%), and the sample based on the PS/C_16_mimCl/Pd(18%) beads and C_16_mimCl dual templates is coded as Pd–SiO_2_(18%).

### Catalysis studies of Pd-loaded hierarchical porous silica

In order to test the catalytic activities of the Pd-loaded hierarchical porous silica (Pd–SiO_2_(12% or 18%)), the catalytic oxidation of 3,3′,5,5′-tetramethylbenzidine (TMB) by H_2_O_2_ was employed as a model reaction. Briefly, in each of glass tubes, 200 μL of TMB aqueous solution (10 mmol L^−1^), 200 μL of H_2_O_2_ solution (250 mmol L^−1^) and 1560 μL of PBS buffer solution (pH = 5) were added with stirring. Then, the Pd-loaded hierarchical porous silica catalysts (1 mg) were added to the above 1960 μL solutions. With constant magnetic stirring, the mixture color changed from colorless to blue. After reacting at different time intervals, the mixtures were scanned by UV-vis absorption spectrograph. The control experiment was also carried out by mixing the solution of TMB and H_2_O_2_ in the absence of the Pd-loaded hierarchical porous silica catalyst. For comparison, the catalytic activities of PS/C_16_mimCl/Pd(12%), PS/C_16_mimCl/Pd(18%), and PS/C_16_mimCl/Pd(24%) beads were studied respectively under identical reaction conditions. According to the amount of PS/C_16_mimCl/Pd bead dispersion used for the synthesis of 1 mg Pd–SiO_2_(12% or 18%), the volume of the bead dispersion tested for the catalytic activity was controlled to be 2 μL. Moreover, the reusability of the prepared Pd–SiO_2_(12%) and Pd–SiO_2_(18%) catalysts were also studied by tracking the changes in UV-vis absorption spectrograph of the reaction system under different cycles.

### Characterization

A field emission scanning electron microscopy (FEI Nova NanoSEM 450) was used to observe the morphology of the samples. Transmission electron microscopy (TEM) images were obtained by using a JEM-2100 electron microscope at an acceleration voltage of 200 kV. The specimens for TEM were prepared by dropping a small drop of the solutions onto a carbon-coated copper grid. Electrophoresis experiments were performed on a Nanjing Sangli DYL-3 electrophoresis apparatus with 15 V of external voltage. X-ray diffraction (XRD) patterns were measured on a TTR III powder X-ray diffractometer using Cu Kα radiation (wavelength 0.154 nm) at a rate of 0.05° 2*θ* s^−1^ and operated at 40 kV and 30 mA. Nitrogen sorption experiments were conducted using a Micromeritics Tristar 3000 automated gas adsorption analyzer. A UV-vis spectrophotometer (SHIMADZU UV-1780) was used to collect absorption spectra of the solution in catalytic reactions. A TENSOR27 Fourier transform infrared (FT-IR) spectrometer was employed for recording IR spectra.

## Results and discussion

### Characterization of C_16_mimCl layer assembled onto PS beads

**Fig. 1:**
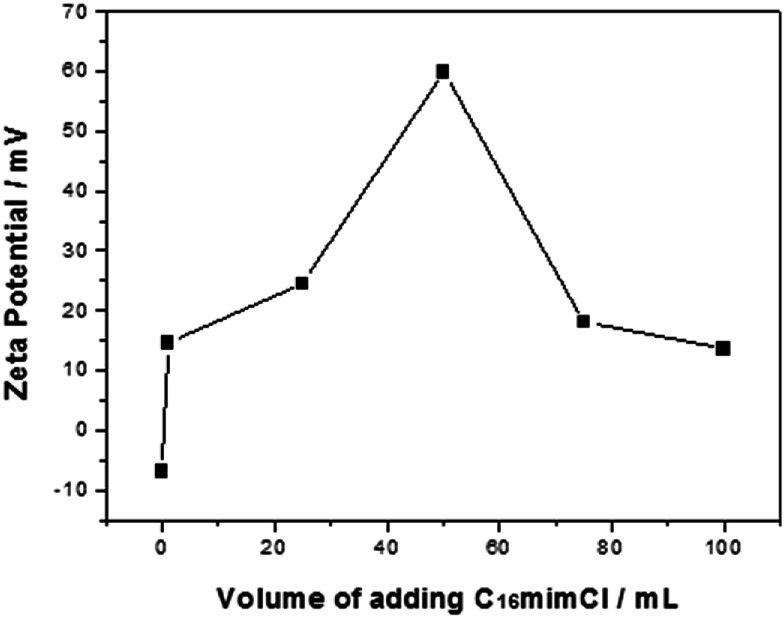
Zeta potential as a function of the volumes of adding C_16_mimCl aqueous solution for the C_16_mimCl-coated PS beads.

PS/C_16_mimCl beads were prepared by adding the C_16_mimCl aqueous solution (2.985 g L^−1^) to PS bead dispersion (4.686 g L^−1^). It was found that a drop by drop and slow adding way for the C_16_mimCl aqueous solution is especially important for preventing the micelle formation of C_16_mimCl in the dispersion. The cmc of C_16_mimCl at 298 K was reported to be 1.21 mM,^28^ and the concentration of one drop of the C_16_mimCl aqueous solution in the suspension is about 0.043 mM, which is well below the cmc of C_16_mimCl. Hence, this adding way is favorable to coat the individual C_16_mimCl molecule onto the PS efficiently. Moreover, we found that C_16_mimCl is freely soluble in water at room temperature, which increases the possibility of the direct interaction between individual C_16_mimCl molecule and PS bead. In order to understand the effect of the amount of C_16_mimCl, different volumes of the C_16_mimCl aqueous solution (*V*(C_16_mimCl) = 1, 25, 50, 75, and 100 mL) were added dropwise and slowly to the PS dispersion (10 mL). Fig. 1 shows that the zeta potential (*ζ*) as a function of the *V*(C_16_mimCl). Two features should be noted. Firstly, the uncoated PS bead dispersion produced a zeta potential of about −7 mV. The presence of a small quantity of C_16_mimCl (1 mL) caused a reversal in zeta potential (+14.5 mV). The multiple tests of adding small quantity of C_16_mimCl, even 0.2 mL of C_16_mimCl, leaded to the zeta potential reversal from negative charge to positive charge, implying a hypothetical structure of the exposure of imidazole head groups of C_16_mimCl toward the surrounding solution, and the hydrophobic long alkyl chain extending to the surface of PS bead. This result showed that the hydrophobic interaction between the PS beads and the alkyl chain of C_16_mimCl is stronger than the electrostatic interaction between the PS beads and the imidazole head groups of C_16_mimCl. Secondly, the increase in the feeding *V*(C_16_mimCl) increases the zeta potential of the resultant PS/C_16_mimCl beads. When the *V*(C_16_mimCl) is 50 mL, the highest value of zeta potential (+60 mV) can be obtained, which is consistent with the value of zeta potential measured from the PS latex particles with poly(allylamine hydrochloride) (PAH) or poly(diallyldimethylammonium chloride) (PDADMAC) as outer shell prepared by LbL method.^29^ The high zeta potential onto the PS/C_16_mimCl beads suggests that a close-packed array of the C_16_mimCl molecules can form onto the PS beads. Sastry *et al.* considered that the increase in the alkyl chain length of ionic liquids leads to the parallel packing of chains in the interior of aggregates and the cationic rings with delocalized π-electron clouds favor high aggregation number.^30^

However, as shown in Fig. 1, further increasing the *V*(C_16_mimCl) to 75 mL, the value of the zeta potential for the PS/C_16_mimCl beads is obviously decreased (18.5 mV). When the *V*(C_16_mimCl) is 100 mL, corresponding zeta potential value becomes 13.6 mV. This is because when the concentration of C_16_mimCl is too high, the excess of C_16_mimCl in the dispersion increase the viscosity of the colloidal solution, and reduce the fluidity of PS/C_16_mimCl beads in the electrophoresis experiments, resulting in the decrease of the zeta potential values. Therefore, we confirm that when the amount of C_16_mimCl is the appropriate value (*V*(C_16_mimCl) = 50 mL), the optimal value of zeta potential (+60 mV) can be obtained.

### Characterization of Pd NPs assembled onto PS/C_16_mimCl beads

**Fig. 2:**
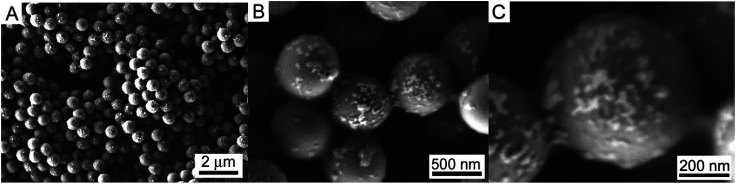
SEM images of PS/C_16_mimCl/Pd(18%) beads with increasing magnification from (A) to (C).

In order to reveal the effect of the amount of Pd NPs, PS/C_16_mimCl/Pd(*x*) beads with *x* = 6%, 12%, 18% and 24% (*x* is different mass ratios of *m*(Pd)/*m*(PS)) were prepared by mixing corresponding volumes (15.62, 31.24, 46.86 and 62.48 mL) of citrate-coated Pd NPs suspensions (0.0599 g L^−1^, *ζ*-potential of −44 mV) with 20 mL of PS/C_16_mimCl dispersion (0.781 g L^−1^, *ζ*-potential of +60 mV), respectively. Fig. 2 shows SEM images of PS/C_16_mimCl/Pd(18%) beads. Compared with the uncoated PS beads with a narrow size distribution and a smooth particle surface (see Fig. S2†), PS/C_16_mimCl/Pd (18%) exhibited a coarse surface in each bead, indicating that a layer of Pd coating with a few clusters was deposited on the surface of PS/C_16_mimCl beads. These SEM images are similar to the images of PS spheres covered with dense and uniform Au NPs.^31^

**Fig. 3:**
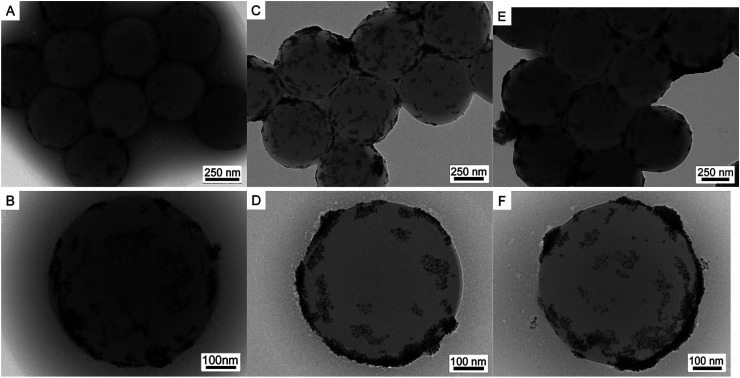
TEM images of (A and B) PS/C_16_mimCl/Pd(12%), (C and D) PS/C_16_mimCl/Pd(18%), and (E and F) PS/C_16_mimCl/Pd(24%).

Fig. 3 further shows TEM images of PS/C_16_mimCl/Pd(*x*) bead with different coverage. Compared to PS/C_16_mimCl/Pd(6%) with an extremely sparse coverage, PS/C_16_mimCl/Pd(12%) (Fig. 3A and B) and PS/C_16_mimCl/Pd(18%) (Fig. 3C and D) exhibited dense and uniform Pd NPs (about 10 ± 3 nm) dispersed onto the C_16_mimCl-functionalized PS beads with a good coverage. It can be found that with the increasing of the mass ratios of *m*(Pd)/*m*(PS) from 18% to 24%, the Pd NPs on the PS beads became more denser (Fig. 3E and F), and some aggregate phenomenon of the Pd NPs in the local position onto PS beads can be observed. This may be because of adding too much Pd NPs, which exceeds the matching quantity of Pd NPs and PS/C_16_mimCl beads. The best matching ratios of the mass ratios of *m*(Pd)/*m*(PS) are 12% and 18%.

**Scheme 1:**
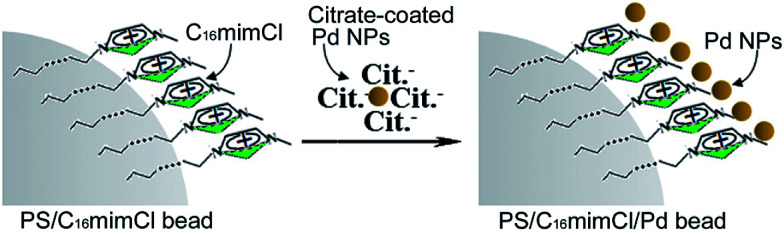
Schematic diagram of the synthesis procedure of PS/C_16_mimCl/Pd beads using C_16_mimCl as a connector.

In brief, there are two steps for the synthesis of the PS/C_16_mimCl/Pd beads. As shown in the Scheme 1, the first step is to get C_16_mimCl-functionalized PS beads by a hydrophobic interaction between the PS beads and the alkyl chain of C_16_mimCl to expose imidazole head-groups of C_16_mimCl molecules to the surrounding solution. In the second step, the PS/C_16_mimCl beads with positive charge can serve as active sites to link the negatively charged citrate-coated Pd NPs by means of an electrostatic interaction. Thus, the C_16_mimCl are applied as ‘bridges’ to connect the PS beads and citrate-coated Pd NPs. It is possible to manipulate the uniformity and coverage of the Pd NPs on the PS beads by controlling the amount of C_16_mimCl and the matching mass ratios of *m*(Pd)/*m*(PS).

### Characterization of Pd-loaded hierarchical porous silica

**Scheme 2:**
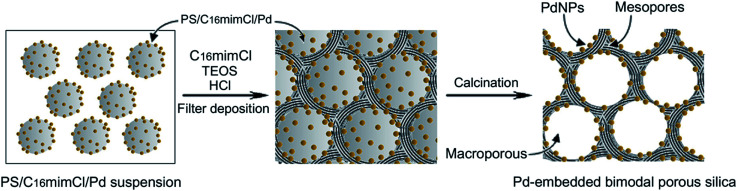
Schematic diagram of the prepared route of Pd-loaded hierarchical porous silica (Pd–SiO_2_) using PS/C_16_mimCl/Pd beads and C_16_mimCl as dual templates.

As shown in the Scheme 2, in the prepared route of Pd-loaded hierarchical porous silica (Pd–SiO_2_), PS/C_16_mimCl/Pd beads were used as a macroporous structure templating agent. The inverse replicas of PS/C_16_mimCl/Pd beads resulted in spherical voids, in which Pd NPs were anchored on the inner walls of the spherical voids. The C_16_mimCl was used as a mesoporous structure templating agent. The inverse replicas of the C_16_mimCl micelles leaded to ordered mesoporous silica channel walls.

**Fig. 4:**
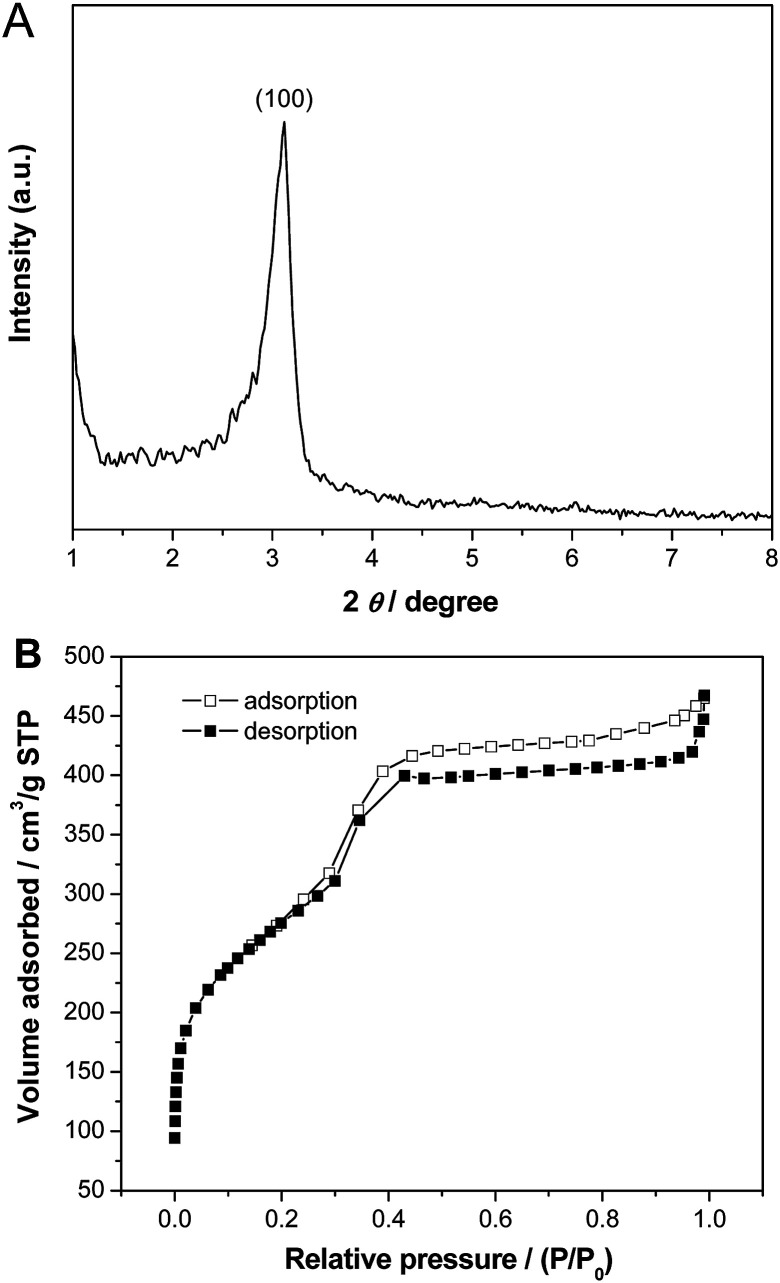
(A) Small-angle XRD pattern and (B) N_2_ adsorption–desorption isotherms of Pd–SiO_2_(12%) prepared using PS/C_16_mimCl/Pd(12%) beads and C_16_mimCl as dual templates.

Fig. 4 shows small-angle XRD pattern and N_2_ adsorption–desorption isotherm of the calcined Pd–SiO_2_(12%) prepared using PS/C_16_mimCl/Pd(12%) beads and C_16_mimCl as dual templates. The sample exhibited one well-resolved diffraction peak (100) in the 2*θ* range between 2 and 6 (Fig. 4A), which is associated with *p*6*mm* hexagonal symmetry (SBA-3 type),^32–34^ indicating a mesoporous structure with short range order. This may be due to a thin wall in the macroporous silica skeleton, thus, only one diffraction peak can be observed. The sample displayed a type IV adsorption isotherm with an obvious hysteresis loop at a relatively high *p*/*p*_0_ values according to IUPAC,^35^ indicating the presence of open pores (Fig. 4B). A steep increasing occurs at a relative pressure 0.30 < *p*/*p*_0_ < 0.43, which is due to the filling of mesoporous walls by capillary condensation. In the previous publication of ordered mesoporous silica structures templated by C_16_mimCl,^15^ the hysteresis phenomenon at relatively high *p*/*p*_0_ values was observed and attributable to the filling of a secondary pore structure, which resulted from grain boundaries or small cavities between adjacent ordered regions. In our sample, the interconnected spherical voids and the Pd NPs onto the walls can be regarded as the grains, and their boundaries with the ordered mesoporous silica channels might create small cavities, therefore, resulting in the more visible and broad hysteresis loop. The BET surface area, total pore volume at relative pressure of 0.98 and mean pore size of the calcined Pd–SiO_2_(12%) are 967 m^2^ g^−1^, 0.68 cm^3^ g^−1^, and 3.0 nm, respectively.

**Fig. 5:**
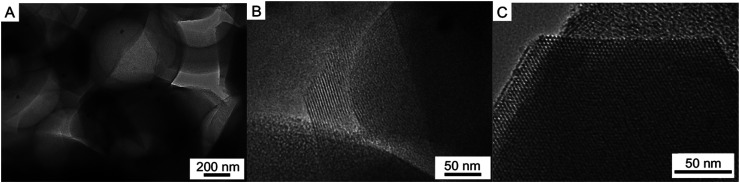
TEM images of (A) Pd–SiO_2_(12%), (B) an enlarged local view of (A), and (C) the local border of the sample.

TEM image of the calcined Pd–SiO_2_(12%) (Fig. 5A) reveals alternating superposition array of the spherical voids (560 ± 10 nm), in which the Pd NPs can be clearly observed with dark particles. A wall of ordered mesoporous channel array (100 direction) (Fig. 5B) can be also observed in the enlarged view of Fig. 5A. However, it should be pointed out that there is only ordered mesoporous silica channel array in the local border of the sample as depicted in Fig. 5C. It can be found that Pd–SiO_2_(18%) has similar TEM images (Fig. S3†), but Pd particle number in the spherical voids increased significantly. These TEM images exhibit direct and conclusive evidence of the coexistence of Pd NPs and well-defined hierarchical porous structures. Furthermore, the small-angle XRD curve of Pd–SiO_2_(18%) appeared some characteristics of cubic phase (Fig. S4†). In classical micelle chemistry, the transformation from hexagonal to cubic mesoporous phase can be explained in terms of the local effective surfactant packing parameter: *g* = *V*/*a*_0_*l*,^36^ where *V* is the total volume of the surfactant chains plus any cosolvent organic molecules between the chains, *a*_0_ is the effective head group area at the micelle surface, and *l* is the kinetic surfactant tail length. When the *g* value is increased above critical values, mesophase transitions occur. For example, a structural change from hexagonal *p*6*mm* to cubic *Ia*3*d* structure should be accompanied by an increase in *g* from 1/2 to 1/2–2/3.^33^ For the synthesis of Pd–SiO_2_(18%), PS/C_16_mimCl/Pd(18%) beads have more Pd particles compared to PS/C_16_mimCl/Pd(12%) beads, which can provide a denser reaction system. Meanwhile, PS/C_16_mimCl/Pd beads possess a certain hydrophobic property, which can be regard as a ‘cosolvent organic molecules’. When the beads are close to the hydrocarbon chains of long-chain ionic liquid, the volume fraction of the hydrocarbon chain should increase. The increase in *V* results in the increase of the value of *g*. Thus, it is possible that the transformation from hexagonal *p*6*mm* to atypical cubic *Ia*3*d* structure can be observed in the small-angle XRD curve of Pd–SiO_2_(18%).

### Catalytic performance of Pd-loaded hierarchical porous silica

**Fig. 6:**
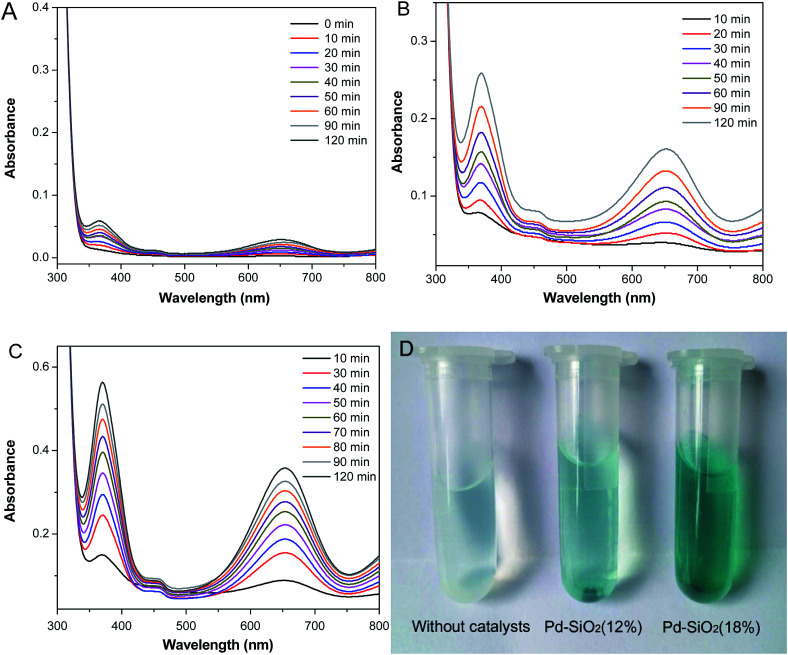
Successive UV-vis absorption spectra of oxidation reaction of TMB by H_2_O_2_ (A) without catalyst, and catalyzed by adding (B) Pd–SiO_2_(12%) and (C) Pd–SiO_2_(18%). (D) Corresponding digital photos of the samples.

**Fig. 7:**
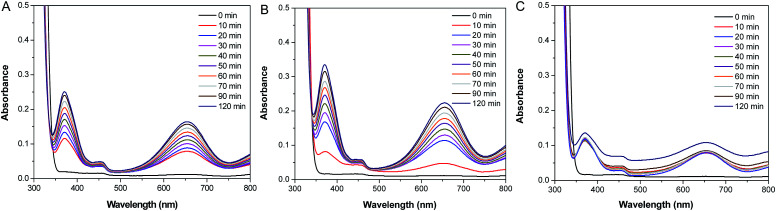
Successive UV-vis absorption spectra of oxidation reaction of TMB by H_2_O_2_ catalyzed by adding (A) PS/C_16_mimCl/Pd(12%), (B) PS/C_16_mimCl/Pd(18%), and (C) PS/C_16_mimCl/Pd(24%) beads.

To evaluate the catalytic activities of Pd–SiO_2_(12%) and Pd–SiO_2_(18%), we employed the oxidation of 3,3′,5,5′-tetramethylbenzidine (TMB) by H_2_O_2_ as a model system. In the previous publication, the oxidation of TMB by H_2_O_2_ can produce diazo dianiline, which exhibited two typical absorption peaks at 370 nm and 652 nm, and induced a color change from colorless to blue in solution.^37,38^ In comparison with the control experiment of the oxidation of TMB by H_2_O_2_ in the absence of catalyst (Fig. 6A), Pd–SiO_2_(12%) and Pd–SiO_2_(18%) used for the oxidation reaction can facilitate the reaction effectively within certain time (Fig. 6B and C), and the catalytic rate of Pd–SiO_2_(18%) is faster than that of Pd–SiO_2_(12%), suggesting that increasing the amount of Pd NPs in the catalyst is beneficial to promote the catalytic performance for the oxidation of TMB by H_2_O_2_. A color change from colorless to blue in the solution (Fig. 6D) can be observed corresponding to the control experiment, the catalytic experiments with Pd–SiO_2_(12%) or Pd–SiO_2_(18%), indicating that Pd–SiO_2_(12%) and Pd–SiO_2_(18%) have enhanced catalytic activity. For comparison, parallel experiments were performed to monitor the catalytic behavior of the synthesized PS/C_16_mimCl/Pd(*x*) beads. Fig. 7 shows the successive UV-vis absorption spectra of oxidation reaction of TMB with H_2_O_2_ catalyzed by adding PS/C_16_mimCl/Pd(12%), PS/C_16_mimCl/Pd(18%), and PS/C_16_mimCl/Pd(24%) beads, respectively. It was observed that the obtained PS/C_16_mimCl/Pd(*x*) beads exhibited significant absorption peaks at 370 nm and 652 nm, indicating that the composite beads had an enhanced catalytic activity compared with the control experiment without catalyst (Fig. 6A). The absorption peak intensity of PS/C_16_mimCl/Pd(12%) beads (Fig. 7A) is similar to that of Pd–SiO_2_(12%) (Fig. 6B). PS/C_16_mimCl/Pd(18%) beads showed higher absorption peak intensity (Fig. 7B) than PS/C_16_mimCl/Pd(12%) beads (Fig. 7A), but had lower absorption peak intensity than Pd–SiO_2_(18%) (Fig. 6C). The high catalytic activity of Pd–SiO_2_(18%) compared to PS/C_16_mimCl/Pd(18%) beads may be attributable to its relatively distinctive hierarchical porous structure. Furthermore, of the three composite beads, PS/C_16_mimCl/Pd(24%) had the lowest absorption peak intensity (Fig. 7C). This may be due to some aggregate phenomenon of Pd NPs in the local position onto PS beads (Fig. 3E and F), leading to the reduction of its catalytic activity.

In the oxidation reaction of TMB by H_2_O_2_, the concentration of H_2_O_2_ was greatly higher than that of TMB, so it is consider as a constant during the reaction process, and the pseudo-first-order kinetics with respect to TMB can be applied as shown by:

1 ln(*c*_0_/*c*) = *kt*

where *c*_0_ is the initial concentration of the TMB solution, *c* is the reactant concentration at time *t* and *k* is the rate constant. Fig. S5† shows a correlation between *A*/*A*_0_ and reaction time for the oxidation reaction of TMB by H_2_O_2_ (*A* and *A*_0_ are absorbance at time *t* and absorbance at the initial stage). For the pseudo first-order kinetics, the *c*/*c*_0_ can be measured from the relative intensity of absorbance *A*/*A*_0_ because *A*/*A*_0_ is in proportion to *c*/*c*_0_. Thus, according to the linear relationship of ln(*A*/*A*_0_) *versus* time (*t*), the rate constant *k* can be calculated. The calculated rate constants are 0.0562 min^−1^ (Pd–SiO_2_(18%)), 0.0436 min^−1^ (Pd–SiO_2_(12%)), and 0.0274 min^−1^ (without catalyst), respectively. It can be clearly seen that the addition of Pd–SiO_2_(18%) and Pd–SiO_2_(12%) significantly fast the oxidation reaction. It is accepted that for the effective promotion of the oxidation reaction, TMB should be concentrated and adsorbed on the surface of the Pd-loaded hierarchical porous silica catalyst due to the high surface area and ordered mesoporous channels. Meanwhile the macroporous spherical voids can increase TMB transport, facilitating access to reactive sites of Pd NPs anchored on the inner walls of the spherical voids. After the completion of the catalytic reaction, the Pd–SiO_2_(12%) and Pd–SiO_2_(18%) catalysts were recovered simply by centrifugation and then dried at room temperature, and finally calcined at 550 °C for 2 h under static air conditions to remove the catalytic reaction products. The recovered catalysts were used again in successive cycles (four times) under identical reaction conditions. Table 2 shows the rate constants of the catalytic oxidation reaction after four cycles. The rate constants were fitted according to the pseudo-first-order kinetics (formula (1)). The results showed that there is no appreciable change of the rate constants for the recovered Pd–SiO_2_(12%) and Pd–SiO_2_(18%) after the three cycles, indicating that there is no loss of catalytic activity. However, after the fourth cycle, the rate constants of the recovered Pd–SiO_2_(12%) and Pd–SiO_2_(18%) were decreased, which suggests their catalytic activity was reduced. The phenomenon may be caused by partial collapse of the hierarchical porous structure of the recycled catalysts.

**Table 2:** Recycling of the catalysts

Sample	Cycle	Rate constant (min^−1^)
Pd–SiO_2_(12%)	1	0.0454
	2	0.0451
	3	0.0435
	4	0.0387
Pd–SiO_2_(18%)	1	0.0543
	2	0.0538
	3	0.0521
	4	0.0468

## Conclusions

In summary, we report a flexible approach for the controllable synthesis of PS/C_16_mimCl/Pd beads using long-chain 1-hexadecyl-3-methylimidazolium chloride (C_16_mimCl) as a connector. The amount of C_16_mimCl and the mass ratios of *m*(Pd)/*m*(PS) were systematically studied to optimize the coverage of Pd NPs. When *m*(Pd)/*m*(PS) = 12% and 18%, the citrate-coated Pd NPs are well-dispersed onto PS beads to form well-defined PS/C_16_mimCl/Pd beads. Moreover, the Pd-loaded hierarchical porous silica with a 3D ordered macroporous skeleton and ordered mesoporous silica walls (SBA-3 type) was prepared using the dual templates of PS/C_16_mimCl/Pd beads and C_16_mimCl. The inverse replicas of the PS/C_16_mimCl/Pd bead template led to well-dispersed Pd NPs anchored on the inner walls of the spherical macroporous voids. Furthermore, the obtained Pd-loaded hierarchical porous silica with a high surface area of up to 967 m^2^ g^−1^ exhibited enhanced and Pd loading capacity-dependent catalytic efficiencies.

## Conflicts of interest

There are no conflicts to declare.

## Supplementary Material

RA-008-C8RA03884H-s001
