# Supplementary material for: Long chain ionic liquid-assisted synthesis of PS/Pd beads and hierarchical porous Pd–SiO2
Source: RSC Adv. 2018 Jul 13;8(44):25141–9. doi: 10.1039/c8ra03884h (PMC9082344; doi:10.1039/c8ra03884h)
Supplement: RA-008-C8RA03884H-s001 [file RA-008-C8RA03884H-s001.pdf]

Electronic Supplementary Material (ESI) for RSC Advances.
This journal is © The Royal Society of Chemistry 2018

Supplementary Material

Long Chain Ionic Liquid-Assisted Synthesis of PS/Pd Beads and Double Porous Pd-SiO_2

Tianlong Wang,[a,b] Ting Fu,[a,b] Yuting Meng,[a,b] Jing Shen,*[a] and Tongwen Wang,[a,b]

[a]*Department of Applied Chemistry, College of Vocational Education, and* [b]*College of Chemistry and Chemical Engineering, Yunnan Normal University, Kunming 650092, China.*

E-mail: shenjingbox0225@hotmail.com

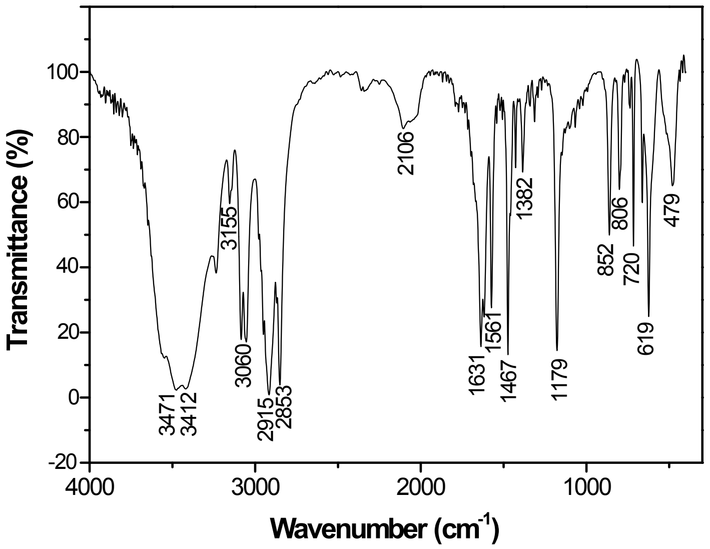

Fig. S1 IR spectrum of 1-hexadecyl-3-methylimidazolium chloride (C_{16}mimCl).

Fig. S1 presents IR spectrum of the synthesized C_{16}mimCl between 400 and 4000 cm^{-1}. The bands at 3471 cm^{-1} and 3412 cm^{-1} can be attributed to the antisymmetric v_3 and symmetric v_1 stretching modes of water, where the water interacts with the anion via H-bonding in a symmetric complex Cl^-···H–O–H···Cl^-(ref. L. Cammarata, S. G. Kazarin, P. A. Salter and T. Welton, *Phys. Chem. Chem. Phys.* 2001, **3**, 5192.). Next to that, the two characteristic bands around 3155 cm^{-1} and 3142 cm^{-1}, which can be assigned to the symmetric v_s CH(4, 5) and asymmetric v_{as} CH(4, 5) stretch of in positions four and five of the imidazolium ring (ref. B. D. Fitchett, J. C. Conboy, *J. Phys. Chem.* 2004, **108**, 20255.). The peaks around 3060 cm^{-1}

are contribution from the asymmetric stretching v_{as} N-CH_3 of the methyl group bound to the imidazolium ring. The next strong bands at 2915 cm^{-1} and 2853 cm^{-1} can be assigned to the antisymmetric v_{as} CH_2 and symmetric v_s CH_2 stretching modes of the alkyl chain. The analysis of other vibration mode assignment can be seen in our early work.[15] Therefore, the results allow conclusion that the C_{16}mimCl with imidazolium ring and alkyl chain was synthesized.

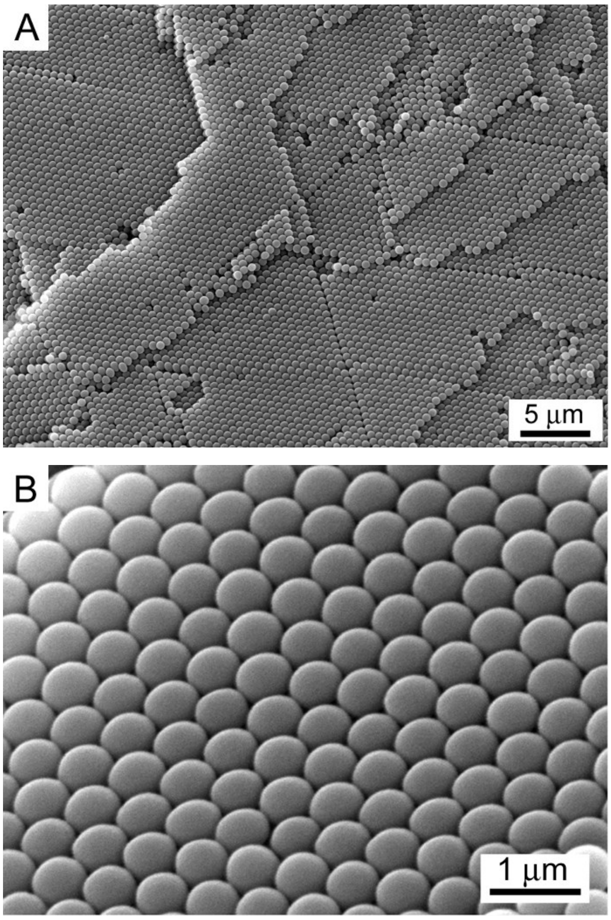

Fig. S2 (A) SEM image of uncoated PS microshperes. (B) is local enlarged position of (A).

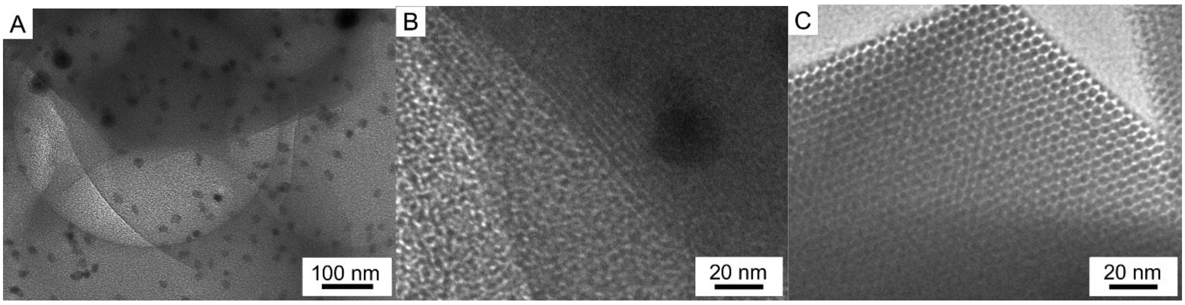

Fig. S3 TEM images of (A) Pd-SiO_2(18%), (B) an enlarged local view of (A), and (C) the local border of the sample.

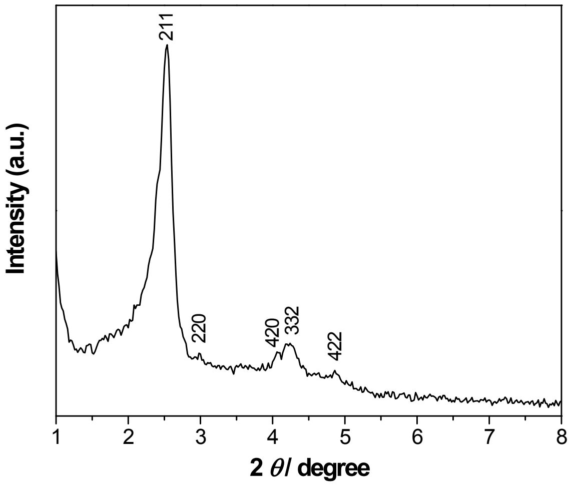

Fig. S4 Small-angle XRD pattern of Pd-SiO_2(18%) prepared using using PS/C_{16}mimCl/Pd(18%) beads and C_{16}mimCl as dual templates.

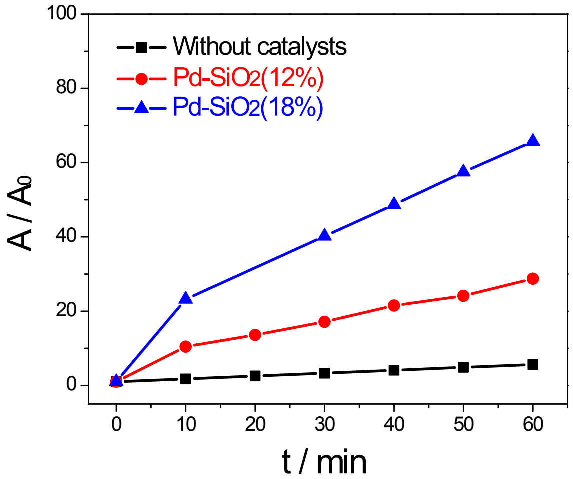

Fig. S5 Plots of A/A_0 against time for the oxidation reactions of TMB by H_2O_2 without and with Pd-SiO_2(12%) or Pd-SiO_2(18%) (A and A_0 are absorbance at time t and absorbance at the initial stage)
